# Aortic Valve Replacement with Rapid-Deployment Bioprostheses: Long-Term Single-Center Results After 1000 Consecutive Implantations

**DOI:** 10.3390/jcm14051552

**Published:** 2025-02-26

**Authors:** Iuliana Coti, Paul Werner, Alexandra Kaider, Jasmine El-Nashar, Alfred Kocher, Guenther Laufer, Daniel Zimpfer, Martin Andreas

**Affiliations:** 1 Department of Cardiac and Thoracic Aortic Surgery, Medical University of Vienna, 1090 Vienna, Austria; paul.werner@meduniwien.ac.at (P.W.); jasmine.el-nashar@meduniwien.ac.at (J.E.-N.); alfred.kocher@meduniwien.ac.at (A.K.); guenther.laufer@meduniwien.ac.at (G.L.); daniel.zimpfer@meduniwien.ac.at (D.Z.); martin.andreas@meduniwien.ac.at (M.A.); 2 Center for Medical Data Science, Medical University of Vienna, 1090 Vienna, Austria; alexandra.kaider@meduniwien.ac.at

**Keywords:** aortic valve replacement, rapid deployment, valve durability, long-term results

## Abstract

**Introduction:** This study aimed to analyze long-term survival and valve-related adverse events after 1000 consecutive rapid-deployment surgical aortic valve replacements (RD-SAVRs) in a single center. **Methods:** A total of 1000 patients following RD-SAVR at our institution were included in a prospective database. Median follow-up was 68 months (IQR: 37–91). Preoperative and operative parameters, survival and valve-related adverse events were assessed. **Results:** Mean age was 73 ± 7 years (45% female). Median EuroSCORE II was 2.7% (IQR: 1.4–5.5). Concomitant procedures were performed in 50% of patients. In the case of isolated SAVR, minimally invasive access was conducted in 415 patients (83%). New early pacemaker implantation was required in 9.1%. Perioperative stroke was observed in 1.6%, and the cumulative incidence of thromboembolic and major bleeding events at 10 years was 8.1% (95% CI: 6.2–10.4%). The 5- and 10-year incidences of severe structural valve degeneration were 0.8% (95% CI: 0.3–2.1%) and 9.2% (95% CI: 4.5–15.9%). Overall re-intervention or re-operation with valve explantation occurred in 38 cases, with a 10-year incidence of 7.7% (95% CI: 5.0–11.2%). Overall 30-day mortality was 0.3% (n = 3) and survival at 1, 5 and 10 years FU was 95% (95% CI: 93–96%), 81% (95% CI: 78–84%) and 58% (95% CI: 51–64%). Age, diabetes, COPD and creatinine, concomitant procedures and acute indication were independent predictive factors of mortality. **Conclusions:** Rapid-deployment valves appear to support minimally invasive access and can be potentially used with low operative mortality in a real-world collective. Favorable durability with acceptable valve-related event rates and mortality were observed at long-term follow-up.

## 1. Introduction

Aortic valve stenosis is the most common valve disease in industrialized countries, with a high prevalence in the elderly population. Symptomatic patients have an unfavorable prognosis and incremental increase in mortality without intervention across the full spectrum of AS severity, suggesting the need for early diagnosis, closer follow-up and earlier treatment of patients [1].

The rapid-deployment Edwards INTUITY Valve System (Edwards Lifesciences LLC, Irvine, CA, USA) is a hybrid surgical aortic bioprosthesis created with the supra-annular Edwards Perimount Magna platform with proven long-term durability and the deployment features of a balloon-expandable transcatheter valve but with the advantage of surgical calcium removal prior to implantation [2,3].

The Edwards Intuity valve demonstrated excellent and stable hemodynamic characteristics in preliminary multicentric trials. Further, in vitro analyses showed decreased flow turbulences in the outflow tract compared with its sutured counterpart, the Edwards Magna Ease [4]. However, bioprosthetic failure is mainly seen at long-term follow-up, and due to the increased number of biological SAVRs in the younger population, a larger number of patients are expected to present with prothesis failure. Further investigation is needed to demonstrate the impact of postoperative valvular hemodynamics on the long-term durability of this surgical bioprosthesis.

Our institution participated in multicentric European trials which demonstrated the safety and efficacy of the rapid-deployment valves, which were standardized at our department with a high volume of implantations and a high number of surgeons using this valve [5].

The aim of this study is to report our long-term, real-world experience with the Edwards Intuity Valve regarding mortality, valve durability and valve-related adverse events after 1000 consecutive RD-AVRs with more than 10 years’ follow-up.

## 2. Materials and Methods

### 2.1. Study Population

Between May 2010 and May 2023, 1000 consecutive patients who underwent surgical aortic valve replacement with an Edwards Intuity Valve System (Edwards Lifesciences LLC, Irvine, CA, USA) at our institution were included in a prospective, ongoing register as previously reported [5] (Figure S1). Patients with severe aortic stenosis or combined disease were included; pure aortic regurgitation and active aortic valve endocarditis were considered contraindications for RD-SAVR. During the study period different bioprostheses were implanted at our institution and device selection was performed according to operator preference (SM). Case selection occurred following our experience in some bicuspid anatomies with very asymmetric annulus, annulus injury during decalcification in order to avoid paravalvular regurgitation or for patients with preoperative conduction disturbances and higher risk for pacemaker implantation, in case of which a conventional sutured prosthesis was preferred. Patients with pure aortic regurgitation, active aortic valve endocarditis or secondary aortic regurgitation due to type A aortic dissection were also implanted with other conventional sutured aortic bio- or mechanical prostheses. The baseline characteristics as well as peri- and postoperative outcomes were assessed in conformity with the AATS/STS/EACTS guidelines for reporting morbidity and mortality after valve surgery [6]. Clinical follow-up was performed at discharge, 1 year and 3, 5 and 10 years after surgery. A telephone follow-up was performed between the clinical visits. Our institutional Ethical Review Board approved this registry (1861/2016).

### 2.2. Study Endpoints

The primary endpoints of this study were long-term durability regarding occurrence of severe structural valve degeneration (SVD) and overall mortality. Severe SVD was defined according to the standardized definition of SVD for surgical and transcatheter bioprosthetic aortic valves [7]. Overall mortality included all deaths after valve implantation regardless of the cause.

Secondary endpoints were the overall rate of re-operations with valve explantations or valve-in-valve (VIV) procedures, reported as a composite endpoint which includes re-operations due to SVD, non-structural valve dysfunction (NSVD) and endocarditis. Another secondary endpoint was the occurrence of thromboembolic and major bleeding events, defined as a composite endpoint which included overall strokes, ischemic transient attacks, peripheral embolism, valve thrombosis and major bleeding events (excluding the surgical re-explorations for bleeding). Postoperative permanent pacemaker implantations (PPIs) were assessed.

### 2.3. Statistical Analysis

Continuous variables are described as mean and standard deviations (SDs) or median and interquartile ranges (IQRs; 25th–75th interval). Categorical variables are reported as total numbers and proportions (in percent).

The inverse Kaplan–Meier method was used to calculate the median follow-up time. The Kaplan–Meier method was performed to assess survival probabilities and the log-rank test was used to test for statistical differences between the curves. Univariate and multivariable Cox models were used to determine potential risk factors for all-cause mortality. The following prognostic factors were included in the multivariable model: gender, age, arterial hypertension, dyslipidemia, diabetes mellitus, chronic obstructive pulmonary disease, perioperative atrial fibrillation, concomitant procedures, elective/acute indication for surgery, BMI, BSA, preoperative creatinine, preoperative LVEF and preoperative NYHA.

The postoperative factors PPI, valve explantation and postoperative occurrence of thromboembolic and major bleeding events were considered as time-dependent factors. All prognostic factors were tested for time-varying effects, which were included in the final model in case of statistical significance.

Trial safety endpoints are reported as early (≤30 postoperative days) or late (>30 postoperative days) events. Early events are reported as numbers and percentages. The probabilities of adverse events during the follow-up were estimated using the cumulative incidence functions accounting for the competing events death (in case of all adverse event outcomes) and valve explantation (in case of the outcomes SVD, valve endocarditis, non-structural valve dysfunction, pacemaker implantation and thromboembolic and major bleeding events, respectively). A multivariable Cox proportional cause-specific hazards regression model was performed to evaluate potential risk factors for the composite endpoint re-operation with valve explantation, considering the prognostic factors age, valve size, preoperative creatinine and gender.

The statistical analysis was conducted using SAS software version 9.4 (SAS Institute Inc., Cary, NC, USA) and SPSS software version 26.0 (SPSS, Inc., Armonk, NY, USA). A 2-sided *p* value of <0.05 was considered to be statistically significant.

## 3. Results

### 3.1. Baseline Characteristics

A total of 1000 consecutive patients underwent SAVR with a rapid-deployment bioprosthesis between May 2010 and May 2023. Follow-up time reached up to 12 years, with a median follow-up of 68 months (IQR: 37–91) and a total accumulated follow-up of 4740 patient years. The mean age was 73 ± 7 years and 45% were female. The median EuroSCORE II and STS Score were 2.7 (IQR: 1.4–5.5) and 1.9% (IQR: 1.3–3.1). Other baseline characteristics are summarized in Table 1.

Concomitant procedures were performed in 500 (50%) patients. More than one attempt for valve deployment to secure the prosthesis in the correct annular position was necessary in 23 (2.3%) cases. Another 17 additional patients, besides the 1000 cases reported, who were no longer followed up, underwent SAVR with a conventional sutured bioprosthesis following one or more failed RD-AVR implantation attempts. Further perioperative data and outcomes are found in Table 2.

### 3.2. Primary Endpoints

#### 3.2.1. Survival

**Figure 1:**
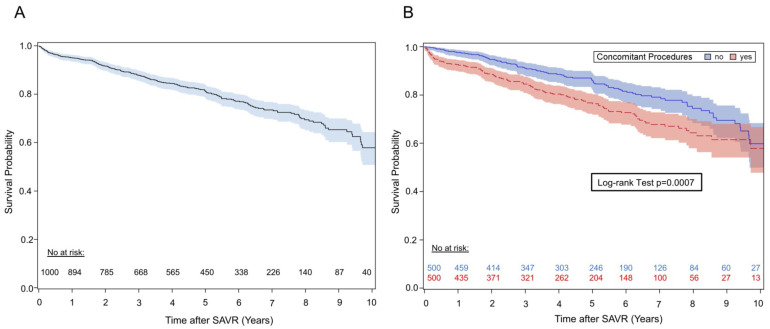
Kaplan–Meier Survival Curve. (**A**) Kaplan–Meier survival analysis; (**B**) log-rank test. Blue = isolated RD-AVR, red = concomitant procedures. The survival in patients with isolated RD-AVR is significantly higher than in patients undergoing concomitant procedures—log-rank *p* < 0.001.

Overall 30-day mortality was 0.3% (n = 3) and 0.2% (n = 1) in isolated RD-SAVR (1/500). Survival at 1-, 5- and 10-year FU was 95% (95% CI: 93–96%), 81% (95% CI: 78–84%) and 58% (95% CI: 51–64%), respectively (Figure 1A). A higher mortality was observed for patients undergoing concomitant procedures (log-rank test *p* < 0.001; Figure 1B).

Univariate and multivariable Cox regression models were created to evaluate pre- and perioperative potential risk factors for all-cause mortality. In this multivariable model, age, diabetes mellitus, COPD, creatinine, concomitant procedures and indication for acute surgery were independent predictors for mortality (Table 3). Additionally, time-dependent postoperative risk factors PPI, valve explantation and postoperative occurrence of thromboembolic and major bleeding events were included along with the abovementioned preoperative and operative factors in a separate multivariable model. In this second model, patients undergoing valve explantation were at higher mortality risk (HR 4.11, 95% CI: 2.30–7.34, *p* < 0.001). Also, patients with a composite endpoint of thromboembolic and major bleeding events presented increased mortality risk (HR 3.64, 95% CI: 2.38–5.56, *p* < 0.001).

#### 3.2.2. Structural Valve Degeneration

**Figure 2:**
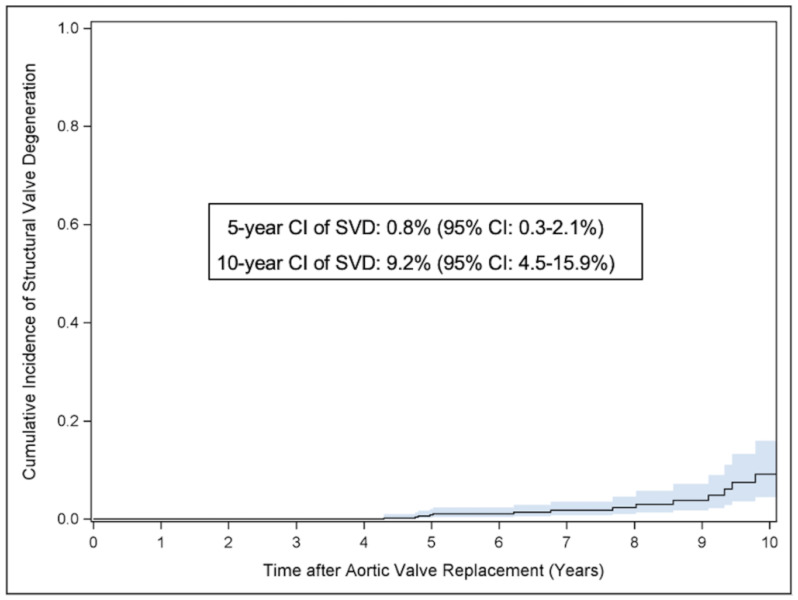
Cumulative incidence curves for severe structural valve degeneration with and without valve explantation. Cumulative incidence of severe structural valve degeneration. Competing risk analysis was performed to estimate the cumulative incidence considering death as competing event.

A total of 14 patients presented with severe SVD with a 5- and 10-year cumulative incidence of 0.8% (95% CI: 0.3–2.1%) and 9.2% (95% CI: 4.5–15.9%). Figure 2 demonstrates the long-term cumulative incidence of severe SVD with and without re-intervention or re-operation on the study valve. However, out of these patients, re-operation or re-intervention on the study valve was performed only in nine patients with a cumulative incidence of 0.6% (95% CI: 0.2–1.7%) at 5 years and 3.6% (95% CI: 1.6–7.0%) at 10 years, Table 4.

### 3.3. Secondary Endpoints

#### 3.3.1. Composite Endpoint Reoperation with Valve Explantation

**Figure 3:**
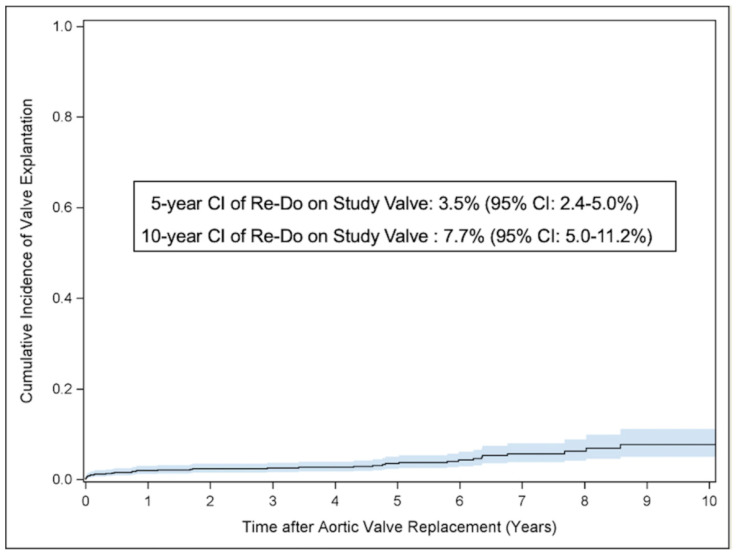
Cumulative incidence curves for composite endpoint re-operation with valve explantation. Cumulative incidence of re-operation with valve explantation. Competing risk analysis was performed to estimate the cumulative incidence of Re-Do after RD-AVR considering death as competing event.

The composite endpoint valve explantation with re-operations due to structural valve degeneration, non-structural valve dysfunction, endocarditis or valve thrombosis occurred in 38 cases with a cumulative incidence at 1-, 5- and 10-year follow-up of 2.0% (95% CI: 1.2–3.0%), 3.5% (95% CI: 2.4–5.0%) and 7.7% (95% CI: 5.0–11.2%); see Figure 3. In a multivariable Cox regression analysis, including age at index procedure, valve size, renal function (creatinine) and gender (male), only age was found to be a significant predictor for re-operation or re-intervention on the study valve, with a higher risk in younger patients (HR: 0.95, 95% CI: 0.91–0.98, *p* = 0.001).

#### 3.3.2. Non-Structural Valve Dysfunction

A total of 75 patients presented with NSVD at the latest follow-up. Moderate–severe paravalvular leak was observed in 25 cases. Re-operation on the study valve due to NSVD occurred in 18 cases with a cumulative incidence at 1-, 5- and 10-year follow-up of 1.4% (95% CI: 0.8–2.4%), 1.8% (95% CI: 1.1–2.8%) and 2.4% (95% CI: 1.4–3.9%).

#### 3.3.3. Valve Endocarditis

A total of 18 patients presented with valve endocarditis at follow-up with a cumulative incidence of 1.8% (95% CI: 1.0–2.9%) and 2.9% (95% CI: 1.7–4.6%) at 5- and 10-year follow-up. Out of these patients 10 patients underwent re-operation with explantation of the study valve, with a cumulative incidence of 1.0% (95% CI: 0.5–2.0%) and 1.7% (95% CI: 0.8–3.1%) at 5 and 10 years.

#### 3.3.4. Permanent Pacemaker Implantation (PPI)

New early pacemaker implantation (≤14 days postoperative) was required in 9.1% of patients. At follow-up, a total of 128 patients required PPI with a CI at 3 months of 10.9% (95% CI: 9.0–12.9%) and a slight increase at 5 years to 13.4% (95% CI: 11.2–15.7%) and 10 years to 15.4% (95% CI: 12.5–18.6%), reflecting the higher perioperative, procedure-related need for permanent pacemaker implantation due to complete AVB. Postoperative PPI was not a significant independent predictor for mortality (HR 1.35, 95% CI: 0.92–1.99, *p* = 0.128).

#### 3.3.5. Composite Endpoint Thromboembolic and Major Bleeding Events

The cumulative incidence of thromboembolic and major bleeding events at 1-year, 5-year and 10-year follow-up was 4.3% (95% CI: 3.1–5.7%), 7.1% (95% CI: 5.5–9.0%) and 8.1% (95% CI: 6.2–10.4%). A total of 64 patients presented with thromboembolic and major bleeding events at follow-up. Valve thrombosis occurred only in one (0.1%) case; perioperative revisions were excluded.

## 4. Discussion

The rapid-deployment Edwards Intuity valve was introduced into clinical practice in 2010 in Europe and 2012 in North America. The first long-term follow-up reports are now providing data with respect to durability.

In this study we report the long-term outcomes after 1000 consecutive implantations with the RD-AV in a single-center setting regarding mortality, re-intervention or re-operation on the study valve and other adverse events.

Williams and colleagues analyzed the long-term outcomes after RD and sutureless AVRs. Four large multicentric studies including the CAVALIER trial, TRITON trial and SURD-AVR registry with a total of 1998 patients were included in a systematic review and meta-analysis. The overall survival rates at 1, 3 and 5 years were 95%, 89% and 84% [8]. The overall survival rate at 5 years in the TRITON study was 81.1% (95% CI: 75.5–86.8%). We report comparable long-term survival rates of 95% and 81% at 1 and 5 years after surgery [9]. At 30 days the TRITON trial showed an overall all-cause mortality of 2.1% (n = 3), compared with 0.8% (n = 7) in the TRANSFORM trial and 0.3% (n = 3) in our cohort of 1000 patients [10,11].

The same meta-analysis published by Williams et al. reports eight cases (0.4%; 95% CI, 0.0–1.1; I^2^ = 73%) of structural valve deterioration (SVD). Endocarditis was reported in 25 patients (1.1%; 95% CI, 0.5–1.9; I^2^ = 55%) and re-operation with valve explantation was observed in 47 patients (2.3%; 95% CI, 1.7–3.1; I^2^ = 7%) [8]. We report an overall cumulative incidence of re-operation or re-intervention on the study valve at 5 and 10 years of 3.5% (95% CI: 2.4–5.0%) and 7.7% (95% CI: 5.0–11.2%); re-operation due to valve degeneration was performed in 0.6% (95% CI: 0.2–1.7%) at 5 years and 3.6% (95% CI: 1.6–7.0%) at 10-year follow-up.

The RD Edwards Intuity Valve was designed with the supra-annular profile and leaflets of the Carpentier Edwards Perimount. Johnston and colleagues described a very low number of valve explantations due to SVD in a large series of 12,569 patients with 81,706 patient years of follow-up after AVR with the Carpentier Edwards Perimount. In this cohort, the overall explantation rate due to SVD was 1.9% and 15% at 10- and 20-year follow-up, respectively [2]. Persson and colleagues described a significantly lower incidence of valve re-intervention in case of the Edwards Perimount valve in a large cohort study including 16,983 patients implanted with different biological aortic prostheses [12]. In this study, the estimated rates of valve explantation at 10 years were 3.6 (95% CI, 3.1–4.2%) for the Edwards Perimount valve, 12.2% (95% CI, 9.8–15.1%) for the Mitroflow and Crown and 11.7% (95% CI, 9.2–14.8%) for the Soprano valve. Bioprostheses with externally mounted leaflets, such as the Mitroflow or the Trifecta valve, showed excellent early hemodynamic performance but significantly increased gradients and SVD at long-term follow-up. Yongue et al. described significantly higher rates of valve explantation at 5 years in a propensity-matched cohort comparing the Trifecta with the Perimount valve [13]. Another analysis conducted at our institution, comparing the Trifecta with the RD Intuity Valve, also demonstrated significantly higher rates of SVD and valve explantation in the Trifecta group [14].

The technical features of RD valves naturally support implantation in minimally invasive settings. In our experience a total of 415/500 isolated AVRs (83%) were operated through minimally invasive access (MIS) with excellent results and a very low access conversion rate (4/415). Other studies confirmed excellent outcomes in MIS RD-AVR. CADENCE-MIS, a prospective randomized study analyzing outcomes after upper hemisternotomy RD-AVR compared with FS AVR with sutured bioprostheses, presented significantly lower cross-clamp times in the RD-AVR cohort (41.3 ± 20.3 vs. 54.0 ± 20.3 min, *p* < 0.001) [15]. Another recent analysis from the SURD-IR described the outcomes after 2257 cases of iAVR with an RD or sutureless prosthesis; as concerns MIS approaches, despite the longer operative times in the ART group (*p* < 0.001), these patients presented with a lower stroke rate (0.04) and significantly reduced intensive care unit (*p* = 0.009) and hospital stay (*p* < 0.001) [16].

One limitation of RD-AVRs might be a higher prevalence of new conduction disturbances associated with permanent pacemaker implantation (PPI). This seems to be related to valve design, which combines a stent-based subannular fixation with a supra-annular prosthesis. Findings from the GARY Registry, including 22,062 patients who underwent iAVR with either an RD (n = 1125) or a sutured aortic valve (n = 20,937), showed higher rates of PPI after RD-AVR (8.8% vs. 3.7%, *p* < 0.001) [17]. In our cohort, the early PPI rate was 9.1% and the occurrence of PPI was not a predictive factor for mortality. Moreover, as reported in a previous study, the presence of baseline conduction disturbances was found to be an independent predictor for PPI [18] and exclusion of patients with previous conduction disturbances might reduce the perioperative rate of PPI after RD-AVR.

Along with emerging innovations in the area of surgical and transcatheter bioprostheses and device selection, medical therapy also evolved and implementation of pharmacological therapies of associated heart failure such as sodium-glucose cotransporter-2 inhibitors (SGLT2is) was associated with reduced all-cause death, MACE and re-hospitalization following invasive treatment of aortic stenosis [19].

## 5. Limitations

This study is an observational study, without center-independent assessment of the adverse events and an independent core lab to assess the echocardiographic and hemodynamic follow-up data. Moreover, this is a single-arm cohort; the aortic valve prosthesis was at the operator’s discretion and the intraoperative decision to select rapid-deployment or other conventional bioprostheses dependent on the anatomy and surgical experience might have occurred during the course of this study.

## 6. Conclusions and Future Research Directions

Rapid-deployment valves might remain a useful tool in the surgical armamentarium as they facilitate minimally invasive access and can be performed with good results and reduced valve-related adverse events. Moreover, our single-center experience after one thousand implantations showed that patient selection might further improve the short- and long-term outcomes following RD-AVR.

## Figures and Tables

**Table 1:** Demographic and baseline characteristics.

Preoperative Patient Characteristics	N = 1000
Age (years, SD)	73.4 ± 7.2
Male (n, %)	548 (54.8)
BMI (SD) kg/m^2^	28.1 ± 5.0
BSA (SD) m^2^	1.9 ± 0.2
EuroSCORE II (%), IQR	2.7 (1.4–5.5)
STS Score (%), IQR	1.9 (1.3–3.1)
NYHA Class III/IV (n, %)	680 (68)
Arterial hypertension (n, %)	876 (87.6)
Dyslipidemia (n, %)	664 (66.4)
Creatinine (mg/dl, IQR)	1.0 (0.8–1.1)
Coronary artery disease (n, %)	444 (44.4)
Cerebrovascular disease (n, %)	189 (18.9)
Peripheral vascular disease (n, %)	86 (8.6)
Chronic lung disease (n, %)	178 (17.8)
Prior atrial fibrillation (n, %)	203 (20.3)
Previous cardiac surgery (n, %)	40 (4)
LVEF (%, SD)	57.6 ± 10.6
Mean transvalvular gradient (mmHg, SD)	52.6 ± 18.1
Peak transvalvular gradient (mmHg, SD)	85.2 ± 29.0
Effective orifice area (cm^2^)	0.73 ± 0.22

BMI = body mass index; BSA = body surface area; SD = standard deviation; IQR = interquartile range; LVEF = left ventricular ejection fraction.

**Table 2:** Operative and perioperative results.

Variables	N = 1000
Elective procedure (n, %)	911 (91.1)
Concomitant procedures (n, %)	500 (50.0%)
Aortic surgery (n, %)	67 (6.7)
CABG (n, %)	319 (31.9)
Mitral valve surgery (n, %)	64 (6.4)
Tricuspid valve surgery (n, %)	41 (4.1)
Atrial fibrillation surgery (n, %)	52 (5.2)
Access (n, %) - Full sternotomy - Upper sternotomy - Thoracotomy	515 (51.5) 252 (25.2) 233 (23.3)
MIS isolated AVR (n, %)	415 (83)
CPB time (min, IQR)	111 (91–140)
XCT time (min, IQR)	77 (60–98)
>1 valve positioning attempt (n, %)	23 (2.3)
Revision for bleeding (n, %)	91 (9.1)
ECMO (n, %)	14 (1.4)
Dialysis (n, %)	18 (1.8)
Early PPI (14-days), (n, %)	91 (9.1)
Perioperative AF (n, %)	257 (25.7)
Perioperative neurological event (<72 h) (n, %)	16 (1.6)
Wound infections (n, %)	27 (2.7)

CABG = coronary artery bypass grafting, CPB = cardio-pulmonary bypass, XCT = cross-clamp time, PPI = permanent pacemaker implantation, AF = atrial fibrillation.

**Table 3:** Preoperative prognostic factors for Long-term Survival (Cox Proportional Hazards Regression Analysis).

	Univariate Analyses	Multivariable Analysis		
Prognostic Factors	HR (95% CI)	*p* Value	HR (95% CI)	*p* Value
Gender (male)	1.12 (0.86–1.47)	0.401	1.07 (0.74–1.54)	0.733
Age (years) *	1.98 (1.61–2.43)	<0.001	1.91 (1.54–2.36)	<0.001
Arterial hypertension	1.17 (0.77–1.77)	0.467	1.07 (0.69–1.68)	0.757
Dyslipidemia	0.96 (0.72–1.26)	0.749	0.86 (0.64–1.61)	0.325
Diabetis mellitus	1.42 (1.07–1.89)	0.016	1.35 (1.00–1.83)	0.049
COPD	1.47 (1.08–2.00)	0.013	1.48 (1.08–2.02)	0.015
Atrial fibrillation	1.50 (1.10–2.05)	0.010	1.12 (0.81–1.55)	0.498
Concomitant procedures		<0.001		0.007
- at 3-month FU	3.36 (1.83–6.17)		2.68 (1.44–4.99)	
- at 5-year FU	1.18 (0.84–1.64)		1.06 (0.75–1.50)	
Elective procedures		<0.001		<0.001
- at 3-month FU	4.79 (2.81–8.15)		3.38 (1.88–6.07)	
- at 5-year FU	1.54 (0.94–2.54)		1.23 (0.72–2.11)	
BMI	0.97 (0.94–1.00)	0.049	1.01 (0.97–1.05)	0.765
BSA	0.56 (0.29–1.08)	0.084	0.47 (0.16–1.42)	0.181
Log_2_ creatinine **	1.94 (1.64–2.31)	<0.001	1.89 (1.54–2.34)	<0.001
LVEF *	0.76 (0.67–0.85)	<0.001	0.89 (0.78–1.01)	0.070
NYHA	1.26 (1.00–1.58)	0.046	0.90 (0.71–1.14)	0.381

Univariate and multivariable Cox regression for survival. * Age unit = 10. * Preoperative LVEF unit = 10. ** Preoperative creatinine (mg/dL) was converted to log_2_ creatinine.

**Table 4:** Long-term Outcomes. Cumulative Incidence Rates at 5 and 10 years.

Variables	N = 1000 CI at 5 Years	N = 1000 CI at 10 Years
Severe SVD (95% CI)	0.8% (0.3 to 2.1%)	9.2% (4.5 to 15.9%)
Re-operation due to SVD (95% CI)	0.6% (0.2 to 1.7%)	3.6% (1.6 to 7.0%)
Re-operation due to NSVD (95% CI)	1.8% (1.1 to 2.8%)	2.4% (1.4 to 3.9%)
Prosthesis endocarditis (95% CI)	1.8% (1.0 to 2.9%)	2.9% (1.7 to 4.6%)
Re-operation due to endocarditis (95% CI)	1.0% (0.5 to 2.0%)	1.7% (0.8 to 3.1%)
Composite aortic valve re-op (95% CI)	3.5% (2.4 to 5.0%)	7.7% (5.0 to 11.2%)
Pacemaker implantation (95% CI)	13.4 (11.2 to 15.7%)	15.4% (12.5 to 18.6%)
Composite thromboembolic– major bleeding event (95% CI)	7.1% (5.5 to 9.0%)	8.1% (6.2 to 10.4%)

SVD = structural valve degeneration, NSVD = non-structural valve dysfunction, composite aortic valve re-op = composite endpoint re-operation and valve explantation or valve-in-valve due to SVD, prosthesis endocarditis or NSVD, CI = confidence interval. The cumulative incidence rates at 5 and 10 years are estimated accounting for death as competing event.

## Data Availability

The data that support the findings of this study are available from the corresponding author upon reasonable request.

## Supplementary Materials

The following supporting information can be downloaded at: https://www.mdpi.com/article/10.3390/jcm14051552/s1, Figure S1: Stented SAVR prosthesis selection.
