# Supplementary material for: Aortic Valve Replacement with Rapid-Deployment Bioprostheses: Long-Term Single-Center Results After 1000 Consecutive Implantations
Source: J Clin Med. 2025 Feb 26;14(5):1552. doi: 10.3390/jcm14051552 (PMC11900265; doi:10.3390/jcm14051552)
Supplement: Supplementary file 1 [file jcm-14-01552-s001.zip › jcm-3426512-supplementary.pptx]

## Slide 1
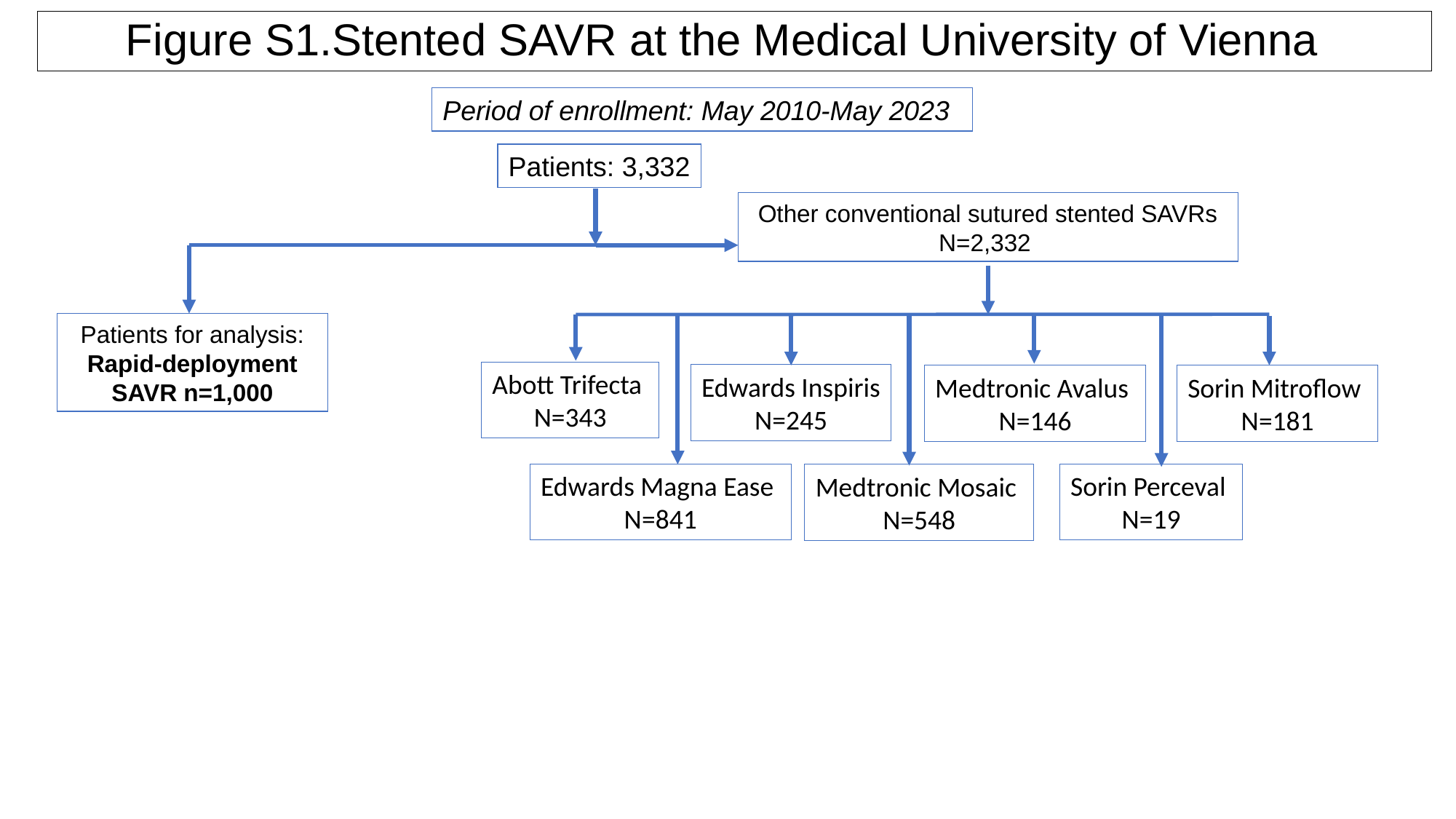

# Figure S1.Stented SAVR at the Medical University of Vienna
Period of enrollment: May 2010-May 2023
Patients: 3,332
Other conventional sutured stented SAVRs N=2,332
Patients for analysis:
Rapid-deployment SAVR n=1,000
Abott Trifecta
N=343
Edwards Inspiris
N=245
Medtronic Avalus
N=146
Sorin Mitroflow
N=181
Sorin Perceval
N=19
Edwards Magna Ease
N=841
Medtronic Mosaic
N=548
